# The Shy Person

**Published:** 1918-06-08

**Authors:** 


### HUMAN TEMPERAMENTS.

### THE SHY PERSON.

The shy person is much to be pitied, for shyness is a curse to its possessor. He suffers all the agonies of remorse without having experienced the pleasure of sinning, so that his shyness is unmitigated misery, and in its more pronounced degrees the shy temperament is a very serious handicap to its possessor in the struggle for life.

We call a shy person self-conscious, and say that he has an exaggerated and hyperæsthetic self-consciousness, but the description, though not altogether false, is not altogether true. It is insufficient and lays undue stress upon one part only, and that not the larger part of the malady. The shy person is unduly self-conscious, it is true, but he is much more other-conscious than self-conscious. His chief trouble is that he is morbidly conscious of other people, of their presence, and above all of their attention to him. To be in the presence of other people, especially of strangers, and in proportion to their strangeness, is to him uncomfortable and embarrassing. To be conscious that they are observing him, that they are paying attention to him, scorches him with agony. And the mischief is that he attributes to them the wish to observe and attend to him, and the fact of observing and attending to him, when they are thinking no more about him than about last year's almanack. He is morbidly self-conscious, it is true, but he is also madly other-conscious, and this is the gravamen of his malady: this is the true source of his misery.

To be embarrassed by knowing that other people are paying attention to us is scarcely abnormal. It is the common lot of humanity. It is an extension and development of that inhibitory effect of all upon each which is the foundation of social life. No one but a lunatic can behave in the presence of other people precisely as he would behave if alone. We can neither eat nor drink, speak nor sing, sit nor walk before other people as we should do if they were not there; and this inhibitory effect that is produced by the mere presence of others is intensified manyfold if we know or think that others are not only present, but are observing and attending to us. The effect is the greater the more numerous the spectators and the stranger they are to us. No one has any difficulty, or feels any embarrassment, in walking across a room. The action is so simple, so easy, so habitual, so automatic from long practice, that it is accomplished without effort and without thought; but take away one side of the room and replace it by the auditorium of a theatre crowded with spectators whose collective attention is concentrated upon you, the sole occupant of the stage, and now do you find it easy and unembarrassing to walk across the room? On the contrary, if you are not accustomed to perform under such conditions, you may find it impossible. In any case you find it very embarrassing, very confusing, and very difficult to perform even the simple act of walking in a natural and easy manner. You have suddenly lost the full power over your limbs. You can move them, it is true, but you have no longer the full, easy, unconstrained command to which you are accustomed. Your movements are awkward and clumsy. You know that they are awkward and clumsy; and the knowledge that they are so, and that many people are witnesses that they are so, embarrasses you still more, and makes them more awkward and more clumsy. What is easier, more natural, less attended with either self-consciousness or other-consciousness than to chat with a friend or two over the fire or across the dinner-table? Who in such circumstances feels any awkwardness or embarrassment, any difficulty in arranging his thoughts or finding words to express them? But let the two or three be multiplied by a hundred, and let the cronies be turned into strangers; how now? If you are not a practised speaker, you will find difficulty, it may be much difficulty. You will gabble as long as words come to you, and when they fail, as they soon will, you will find your tongue paralysed, and, if words should at length come to you, you will be unable to utter them. The room swims before you. The sea of faces blends into one undistinguished blur. It needs only a titter to make you sink into your shoes or rush from the platform. If you have had on exceptional occasion such experiences as these, you have some taste of what the shy person suffers, not on exceptional occasion, but whenever he is where two or three are gathered together. What you feel with moderate intensity only when the attention of a considerable audience is concentrated upon you, he feels with great intensity whenever he is in the presence of others who might be observing him and attending to him, whether they are actually doing so or not. As in some people the eye is morbidly over-sensitive to light, and in others the ear is morbidly over-sensitive to sound, or the skin morbidly over-sensitive to touch, so in the shy man the consciousness is morbidly over-sensitive to attention. It is one great raw, and the lightest touch upon it is agony.

The effect upon us of even the presence of other people, and still more of the knowledge that they are paying attention to what we do and say, is always inhibitory—that is to say, it checks, hinders, limits our activity. It is inexplicably paralysing. Pushed to excess, it becomes actually paralysing. It not only checks and limits our activity, both of body and of mind, but almost abolishes it. The mind becomes confused, obscured, embarrassed, and acts but slowly and erratically, or scarcely acts at all; and the body faithfully reflects the state of the mind. Movements are tardy, awkward, and fumbling. Speech becomes difficult, disconnected, inappropriate to the occasion, even incoherent. This is what we may all suffer if the attention of many strangers is concentrated upon us; this is what the shy person suffers if the attention of even a few strangers, if the attention of but a single stranger, is concentrated upon him. And the effect is intensified in a vicious circle. The sufferer is

**HUMAN TEMPERAMENTS**—(*continued*).

acutely conscious of his awkwardness and embarrassment, and the more he is conscious of them the more they increase; the more they increase the more acute and painful his consciousness of them becomes, until at length in extreme cases he bursts into tears or rushes from the room in panic and despair.

The embarrassment of mind is reflected and expressed not only in conduct, but in organic changes also, and the chief of these, the proclamation of shyness and embarrassment to all spectators, is the phenomenon of blushing. Blushing proclaims to the spectators the shyness and embarrassment that are felt in consequence of their attention, thereby attracts their attention and increases it, and thereby are themselves increased. The unhappy shy person who proclaims his shyness by blushing enhances his own misery fivefold.

No one but the shy person who blushes can appreciate or understand his misery. It is so acute, and seems so incurable that the consequences are serious. A shy person has been known to be exasperated by his shyness to the point of suicide itself, and though such extreme exasperation as this is rare, it is not at all rare, nor is it at all to be wondered at, that the shy person avoids the circumstances and occasions in which he experiences shyness. In other words, he shuns every occasion in which attention would be attracted to himself. He shuns publicity. He keeps himself private. He shuns meeting strangers. He narrows his acquaintances. He lives more and more to himself and by himself; and as he has less and less experience of meeting strangers, less and less opportunity to experience their attention and become accustomed to it, he becomes more and more confirmed in his habit of shyness, which becomes more and more difficult to break. Such is one of the consequences of shyness; but there is an alternative.

Shyness is sometimes interpreted as cowardice, but it is not necessarily accompanied by cowardice. Of what is called physical courage—that is to say, of the power to meet physical danger with a steady eye and hand, the shy man may have abundance. Fear of physical danger, which is what is usually meant by physical cowardice, he may be completely free from. In the presence of physical danger he may be as brave as a lion. If by moral courage is meant the ability to meet with steady mind the disapprobation of his fellows, he may possess this also; but he certainly does not possess the kind of courage that enables him to bear with equanimity the glare of their attention; and he feels that, justly or unjustly, this inability may bring upon him the disesteem and reproach that attach to the coward. If he takes this view, and if he is in fact a courageous man, he will be apt to make some display in order to repel the imputation. Or it may be that being, as the shy man always is, acutely sensitive to this defect, and as the shy man not always is, determined to overcome it, to ignore it, and to behave as if it did not exist, he endeavours by active efforts to show that he is not embarrassed. He will not display his embarrassment, not he. He will cover it up and disguise it beneath a display of ease and self-possession; and so determining, he overacts the part. The embarrassment that he actually feels, in spite of the pretence that he does not, confuses his mind and impairs his judgment, so that instead of a display of ease and nonchalance he becomes boisterous, rude, audacious, ill-mannered, and lays up for himself an additional agony of remorse, to be experienced when the occasion is over, and he reflects in cool blood upon what he has done.

What, then, is the remedy for shyness? What is the shy man to do in order that he may be shy no longer? How is this failing to be overcome? The remedy is simple, and is to be found by consideration of the cause. The shy person is shy in the presence of strangers only. Among his familiars he experiences no shyness. With those who are no strangers to him he feels no shyness and he is shy in proportion to the amount of attention from strangers that is concentrated upon him; and, in less degree, but still in some degree, to the strangeness of the occasion. This being borne in mind, the means of prevention is obvious. Let him have no opportunity of meeting strangers, and let the opportunity be abolished not by abolition of the meetings, but by abolition of the strangeness. In other words, shy persons are those who in early life have had no practice and no experience in meeting strangers, and so having the attention of strangers directed to them and attracted to them. Something is due to innate constitution, no doubt. All children are shy when young, but some are born more shy than others; but, born shy or not, shyness can be prevented by accustoming children from an early age to meet all kinds of people. By custom strangeness is abolished. Custom and strangeness are incompatible, and in proportion as custom prevails strangeness is driven away. And shyness depends largely upon strangeness. Not wholly, for a number of strangers accentuates shyness; but consciousness of the attention of strangers is at the bottom of it. If the meeting with strangers becomes customary it loses its strangeness. The strangers are individually still strangers, but the occasion of meeting strangers is no longer strange, and when the strangeness is abolished, the shyness is abolished along with it.

What suffices for prevention suffices for cure also. Cure is much more difficult than prevention, and therefore no child should be allowed to grow up in the habit of shyness; but cure may always be effected by the same means that prevention can be effected—that is, by the shy person accustoming himself to occasions on which he experiences shyness. He will go through agonies in the process, and the cure will take longer the older he is before he begins to practise it; but if taken in hand at any reasonably early age, the cure is certain. It needs courage. It needs grit and determination. It takes time. It may take years. But one fine day the shy man will wake to the delightful consciousness that his shyness is vanished.

